# Evidence of Mercury Methylation and Demethylation by the Estuarine Microbial Communities Obtained in Stable Hg Isotope Studies

**DOI:** 10.3390/ijerph15102141

**Published:** 2018-09-29

**Authors:** Neusa Figueiredo, Maria Luísa Serralheiro, João Canário, Aida Duarte, Holger Hintelmann, Cristina Carvalho

**Affiliations:** 1 Research Institute for Medicines (iMed.ULisboa), Faculty of Pharmacy, Universidade de Lisboa, Av. Prof. Gama Pinto, 1649-003 Lisboa, Portugal; nfigueiredo@ff.ulisboa.pt (N.F.); aduarte@ff.ulisboa.pt (A.D.); 2 BioISI—Biosystems & Integrative Sciences Institute, Faculty of Sciences, Universidade de Lisboa, Campo Grande C8, 1749-016 Lisboa, Portugal; mlserralheiro@fc.ul.pt; 3 Centro de Química Estrutural, Instituto Superior Técnico, Universidade de Lisboa, Av. Rovisco Pais 1, 1049-001 Lisboa, Portugal; joao.canario@ist.utl.pt; 4 Chemistry Department, Trent University, 1600 West Bank Drive, Peterborough, ON K9J 0G2, Canada; hhintelmann@trentu.ca

**Keywords:** bacteria, SRB, estuaries, methylmercury, mercury, methylation, demethylation, biogeochemistry, estuarine chemistry, mercury isotopes

## Abstract

Microbial activity is a critical factor controlling methylmercury formation in aquatic environments. Microbial communities were isolated from sediments of two highly mercury-polluted areas of the Tagus Estuary (Barreiro and Cala do Norte) and differentiated according to their dependence on oxygen into three groups: aerobic, anaerobic, and sulphate-reducing microbial communities. Their potential to methylate mercury and demethylate methylmercury was evaluated through incubation with isotope-enriched Hg species (^199^HgCl and CH_3_^201^HgCl). The results showed that the isolated microbial communities are actively involved in methylation and demethylation processes. The production of CH_3_^199^Hg was positively correlated with sulphate-reducing microbial communities, methylating up to 0.07% of the added ^199^Hg within 48 h of incubation. A high rate of CH_3_^201^Hg degradation was observed and >20% of CH_3_^201^Hg was transformed. Mercury removal of inorganic forms was also observed. The results prove the simultaneous occurrence of microbial methylation and demethylation processes and indicate that microorganisms are mainly responsible for methylmercury formation and accumulation in the polluted Tagus Estuary.

## 1. Introduction

Methylmercury (MeHg) is one of the most toxic forms of mercury [1] and has been extensively studied for its neurotoxic effects such as blindness, loss of balance, and in severe cases, death [2]. Humans are mainly exposed to MeHg via the consumption of fish and marine mammals [3]. The release of mercurial compounds by industrial activities has been the cause of two large epidemic disasters related to the consumption of contaminated fish in Japan (Minamata Bay and Agano River) [3].

The Tagus Estuary (Portugal), the object of this study, experienced high levels of mercury contamination as a result of past industrial activity mainly related to pyrite processing and chloralkali production [4,5,6]. In particular, the two areas included in this study in the North (Cala do Norte) and the South (Barreiro) are severely contaminated, with reported levels for total mercury of up to 11 and 33 mg/kg and for MeHg of up to 28 and 47 µg/kg, respectively [7].

MeHg is formed in the environment when the oxidized mercury species react with a methyl group [8]. Methylation of mercury can occur under aerobic and anaerobic conditions by abiotic or biotic mechanisms. The abiotic transmethylation reactions include the transfer of a methyl group by the action of ultraviolet radiation or by reaction with humic and fulvic acids [9]. However, in aquatic environments, the biomethylation of mercuric mercury is the major pathway responsible for high concentrations of MeHg [10]. The biomethylation of mercury has been associated with a wide array of organisms with different metabolic pathways and taxa [11]. It was first described by Jensen and Jernelov in 1969 [12], who at the time assumed that the methyl group was transferred to Hg^2+^ by the carbon monoxide dehydrogenase (CODH) pathway in sulphate-reducing bacteria (SRB) [8]. Therefore, the production of MeHg was primarily associated with SRB [13]. Later, iron-reducing bacteria [14] and methanogens [15] were also associated with MeHg production in anoxic environments. Recently, a genetic basis for mercury methylation was provided through the identification of two genes (*hgcA* and *hgcB*) in mercury-methylating bacteria [16].

Numerous microorganisms have been also reported to convert MeHg into less toxic forms [17] by cleaving the carbon-mercury bond (Hg–CH_3_) [18]. Both oxidative and reductive demethylation pathways have been reported. Oxidative demethylation is mediated by anaerobic bacteria and probably related to carbon metabolism (C1), with release of CO_2_ and Hg^2+^ [1]. Reductive demethylation leads to the formation of Hg^0^ and CH_4_ as end products and is usually genetically encoded by a cluster of genes organized in the *mer* operon [8]. The encoded enzymes break the covalent Hg–C bond (organomercurial lyase) and reduce Hg^2+^ to Hg^0^ (mercuric reductase) [19,20,21]. However, abiotic MeHg degradation is also possible, such as the photodegradation of MeHg mediated by the action of ultraviolet light [8].

The concentration and bioaccumulation of MeHg in aquatic environments depends on the balance between both methylation and demethylation processes. In the aquatic environment, these two processes are typically microbially mediated and occur mostly in sediments [10,22]. The rate of these processes is a function of the microbial activity and the concentration of bioavailable mercury species. Thus, the integrated study of these two simultaneous processes is important to understand the dynamics of production and degradation of MeHg, which is critical for future remediation management in contaminated environments.

These processes have been investigated using sediments and pure cultures of isolated microorganisms under anaerobic and aerobic conditions [14,15,22,23,24,25]. The objective of the present work was to investigate communal activities of bacteria and establish their contribution for the processes of mercury methylation and demethylation by using isotope-enriched Hg species and inductively coupled plasma mass spectrometry (ICP-MS) analysis.

## 2. Materials and Methods

### 2.1. Studied Areas and Sampling

**Figure 1:**
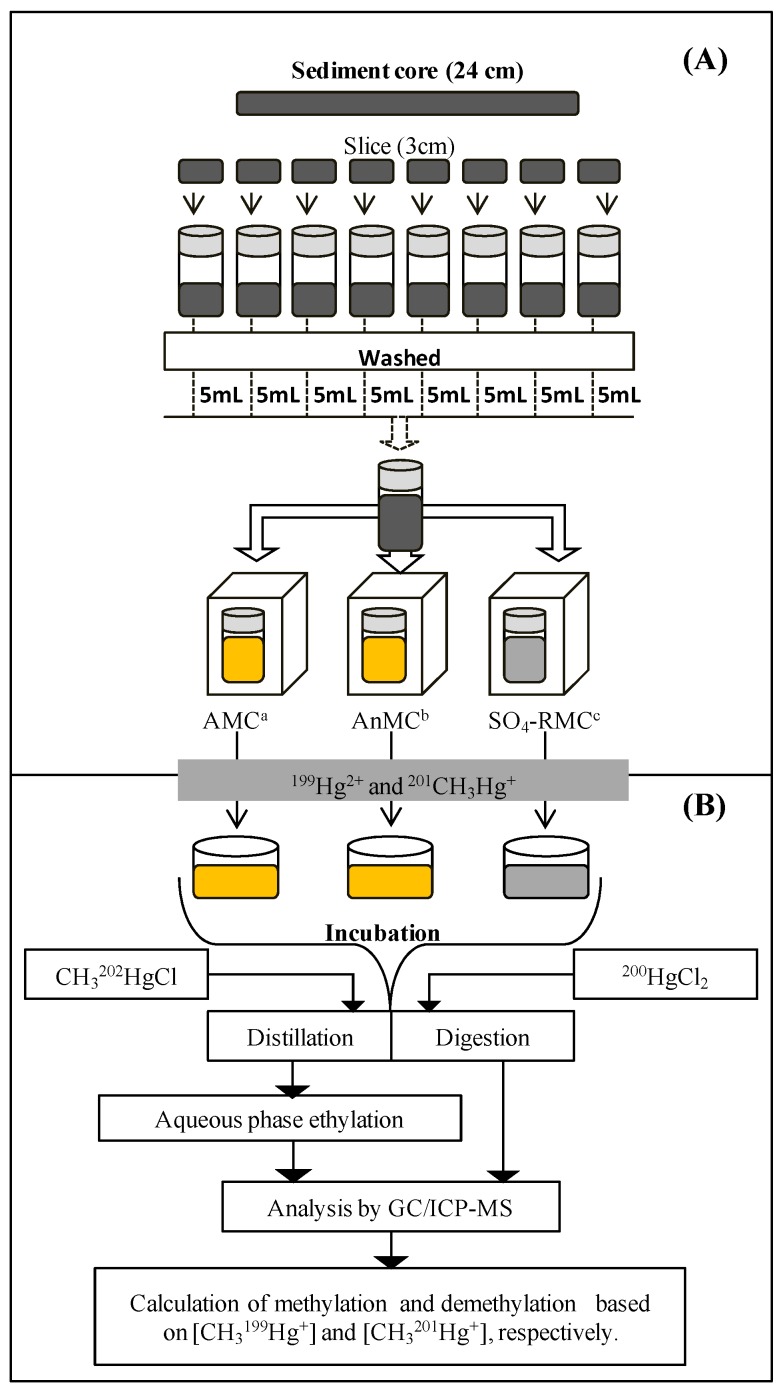
Illustration of the process for the isolation of microbial communities (**A**) and subsequent incubation with isotope-enriched Hg species to evaluate microbial potential to methylate and demethylate mercury (**B**). CH_3_^201^Hg degradation and CH_3_^199^Hg production were monitored using gas chromatography and inductively coupled plasma mass spectrometry (GC/ICP-MS) analysis.

Two areas of the Tagus Estuary were sampled: Barreiro—38°40′45.40″ N; 9°3′1.70″ W and Cala do Norte—38°51′21.21″ N; 9°3′40.51″ W. Sediment samples were collected during spring. Sediment cores of approximately 24 cm in length were collected and rapidly sliced into layers of 3 cm (Figure 1). Samples were stored refrigerated in sealed tubes and transported to the lab for mercury-resistant microbial community isolation.

### 2.2. Microbial Communities’ Isolation

Inoculums were prepared through the dilution of sediment samples with 20 mL of distilled and sterile water. After vigorous shaking, 5 mL was taken from each suspension and added to a new tube, creating a mixture of the 24-cm sediment core (Figure 1A). The mixture was shaken and after centrifugation at 5000 rpm for 1 min (4 °C), 2–5 mL of supernatant was inoculated into liquid media containing 2 µg/mL Hg^2+^. Figure 1A schematizes the techniques used for the isolation of different Hg-resistant microbial communities: the aerobic microbial community (AMC), anaerobic microbial community (AnMC), and sulphate-reducing microbial community (SO_4_-RMC), which are also described below.

***Aerobic community***: To isolate the AMC, 2 mL of washed sediment supernatant was inoculated in 20 mL of Mueller–Hinton (MH) broth and incubated under aerobic conditions. After 24 h of growing at 37 °C, bacterial growth was visible. From this bacterial suspension, 2 mL of the inoculums were transferred to a new MH broth containing 2 µg/mL Hg^2+^ and incubated in aerobic conditions.

***Anaerobic communities***: To isolate the AnMC and SO_4_-RMC, 5 mL of the supernatant was inoculated in serum bottles (Belco Glass Inc., Vineland, NJ, USA) containing 50 mL of MH broth and *Postgate* C medium, respectively. (*Postgate* C contains sulphate that can be reduced to sulphide, forming a black precipitate that indicates SRB growth [7]). Media were prepared under nonsterile conditions and added to N_2_-gassed serum bottles and closed with rubber stoppers with a crimped metal seal, after which the bottled media was autoclaved. To avoid O_2_ contamination, all inoculations were performed using anaerobic techniques under N_2_ flux in an anaerobic glove box. After 3 days of growing at 37 °C, bacterial growth was visible, and 5 mL of the inoculum was transferred to new bottled medium supplemented with 2 µg/mL Hg^2+^.

All three communities were stored in the respective media (MH broth or *Postgate* C) plus 15% of glycerol containing 2 µg/mL Hg^2+^ at −80 °C.

### 2.3. Determination of Mercury Resistance

Mercury resistance levels of each microbial community were determined, as described previously for individual bacteria [26] for mercuric mercury (HgCl_2_) ((Sigma-Aldrich, St. Louis, MO, USA Portugal),) and MeHg (CH_3_HgCl) (Sigma-Aldrich, St. Louis, MO, USA), using nominal concentrations ranging from 0.01 to 1003 µg/mL Hg^2+^ and 0.01 to 100 µg/mL CH_3_Hg^+^. Determinations of mercury resistance were carried out in duplicate at each concentration tested. After incubation at 37 °C for 24 h in the dark and under aerobic and anaerobic (anaerobic jars with AnaeroGen sachet (Oxoid, Basingstoke, UK)) conditions, bacterial growth was monitored. The mercury resistance was registered as the lowest concentration of test compounds without visible growth. All data points represent the mean ± standard deviation (STD) of 2 independent determinations (each one also performed in duplicate).

### 2.4. Mercury Methylation and Demethylation Evaluation

Methylation and demethylation potential were evaluated simultaneously for the three isolated microbial communities as illustrated in Figure 1B. A spike solution containing isotope-enriched ^199^HgCl_2_ and CH_3_^201^HgCl in a proportion of approximately 100:1 was prepared (see below 2.5 and added to the growth media, where the microbial communities (AMC, AnMC, and SO_4_-MC) were placed. After incubation, MeHg analysis was performed as described below.

### 2.5. Preparation of the Spike Solution

A stock solution of ^199^HgCl_2_ (880 µg/mL ^199^Hg) was obtained by dissolving ^199^Hg-enriched (91.95% purity) HgO (Oak Ridge National Laboratories) in 1 mL of hydrochloric acid (10 mM). CH_3_^201^HgCl (96.17% purity) used for the demethylation assay was synthesized from HgO (Oak Ridge National Laboratories, Oak Ridge, EUA) using the methylcobalamin method. The spike solution was prepared by adding 60 µL of ^199^Hg stock and 15 µL of CH_3_^201^HgCl (80 µg/mL) to deionized water (final volume of 5 mL). Thus, the spike solution contained 0.205 µg/mL of CH_3_^201^Hg and 10.56 µg/mL of ^199^Hg and was used for the subsequent methylation and demethylation assays.

### 2.6. Microbial Community Incubation with Mercury Isotopes

Mercury spike solution was added to overnight culture suspensions (adjusted to 10^6^ colony-forming units (CFU)/mL) to achieve 0.106 µg/mL of ^199^Hg and 0.002 µg/mL of CH_3_^201^Hg. The microbial community suspensions and controls (MH broth and *Postgate* C medium plus spike solution) were incubated at 37 °C under aerobic conditions for AMC and anaerobic conditions for AnMC and SO_4_-RMC. Anaerobic conditions were achieved using serum bottles prepared as described above (Section 2.1 of Material and Methods). Samples were taken after 6 and 28 h of microbial growth, and in the case of SO_4_-RMC, also at 48 h. After each experimental endpoint, the optical density was measured using absorption spectrophotometry (595 nm) and the microbial suspension was filtered using syringe 0.4-µm filters (Acrodisc, Sigma Aldrich, Merck KGaA, Darmstadt, Germany) to separate the supernatant for further methylmercury analysis. Two independent experiments were carried out for each experimental condition.

### 2.7. Analysis of MeHg

The analysis of MeHg was performed via distillation/ethylation. MeHg was extracted from supernatant samples using water vapor distillation. Supernatant aliquots (250 µL) were transferred into Teflon distillation vials containing 10 mL deionized water, 200 µL KCl (20% *v*/*v*), and 500 µL H_2_SO_4_ (9 M). The samples were distilled under a nitrogen gas flow of 80 mL/min at 135 °C. The distillate was collected into Teflon distillation vials containing 5 mL of deionized water. After collection of approximately 90% of the distillate, the process was stopped. Blanks were prepared following the same procedure. Total MeHg was measured on a Tekran 2700 MeHg Auto Analysis System, using EPA method 1630 developed by US Environmental Protection Agency. 

The concentration of isotopes (CH_3_^201^Hg and CH_3_^199^Hg) was quantified after gas chromatographic separation using inductively coupled plasma mass spectrometry (X-Series II ICP-MS, Thermo Fisher Scientific Inc., Waltham, MA, USA) by species-specific isotope dilution, using CH_3_^202^HgCl as an internal standard, which was added to each sample before the distillation (Figure 1B). The measurement procedure and the scheme to calculate the tracer concentrations are described in detail elsewhere [27]. The following isotopes of Hg were measured: ^199^Hg (Hg methylation), ^201^Hg (MeHg demethylation), ^202^Hg (internal standard), and ^200^Hg (representing ambient MeHg). For each batch of samples, distillation blanks were analyzed for quality control purposes and found to be negligible (0.9 ± 0.9 pg per distillation), resulting in typical detection limits of 0.011 ng/mL. MeHg recovery was typically >80%.

### 2.8. Analysis of Total Hg

Total mercury was determined in digested samples of supernatants using cold-vapor atomic fluorescence spectrometry (CV-AFS). Filtered samples were treated overnight with an oxidant agent (0.5% of 0.2 N bromine monochloride solution, BrCl) plus 0.5% HCl to convert all mercury into its ionic form. The digestion was stopped with the addition of NaH_2_OH·HCl (20 µL to 40 mL). To correct for procedural losses, ^200^HgCl was added to the samples as an internal standard prior to the digestion (Figure 1B).

The concentration of Hg isotopes in the digest was quantified using continuous-flow cold-vapor ICP/MS analysis (X-Series II ICP-MS, Thermo Fisher Scientific Inc.). The acidified sample was continuously mixed with a solution of stannous chloride 3% (*w*/*v*) in 10% HCl (*v*/*v*) by means of a peristaltic pump. The formed mercury vapor was separated from the liquid using an in-house-made gas–liquid separator and the elemental mercury was swept into the plasma of the ICP/MS. The following isotopes of mercury were measured and quantified using isotope dilution calculation: ^199^Hg (from ^199^HgCl added for methylation essay), ^201^Hg (from CH_3_^201^Hg added for demethylation essay), ^200^Hg (internal standard), and ^202^Hg (to calculate ambient total mercury). For each batch of samples, digestion blanks were analyzed for quality control purposes and found to be negligible (0.15 ± 0.1 ng/mL), resulting in typical detection limits of 0.48 ng/mL for a 50-mL sample.

### 2.9. Determination of Methylation and Demethylation Rates

The formation of MeHg was evaluated by measuring the amount of MeHg production (CH_3_^199^Hg) from the inorganic spike (^199^Hg), and the rate was calculated as: Methylation (%) = ([CH_3_^199^Hg]_Final_ × 100)/[^199^HgT]_Initial_. The percentage of demethylated CH_3_^201^Hg was calculated as follows: Demethylation (%) = ([CH_3_^201^Hg_Initial_ − CH_3_^201^Hg_Final_] × 100)/[CH_3_^201^HgT]_Initial_.

### 2.10. Evaluation of Microbial Hg-Reduction Potential

Mercury reduction and subsequent volatilization of Hg^0^ was verified according to the protocol described by François et al. [28], with some modifications. To the overnight microbial community adjusted to 10^6^ CFU/mL with MH in a 12-well microplate, HgCl_2_ solution was added to achieve 2 µg/mL Hg^2+^. A sensitive X-ray film layer was inserted in the microplate, followed by incubation at 37 °C in the dark for 48 h. The Hg^0^ volatilization was observed through the foggy areas on the X-ray film, due to the reduction of Ag^+^ by mercury vapor (Hg^0^). The optical density was measured at 595 nm (Hitachi spectrophotometer), and the cells were harvested by centrifugation at 15,300 × *g* for 5 min with the supernatant and cell pellet separated for total mercury (HgT) analysis. Harvested cells were washed with sterile deionized water and weighed.

Determination of HgT was performed by pyrolytic reduction and atomic absorption spectrometry using a LECO AMA-254 gold amalgamator [29]. The experiment was performed in duplicate with an uninoculated control run in the same conditions. The percentage of reduction was calculated as: Reduction (%) = (HgT_Initial_ − (HgT_Supernatant_ + HgT_Cell pellet_))/(HgT_Initial_) × 100.

## 3. Results

### 3.1. Microbial Community Characterization 

Sediments from two mercury-polluted areas of the Tagus Estuary Barreiro and Cala do Norte were sampled (Figure 1) to isolate three microbial communities exhibiting mercury resistance: the aerobic microbial community (AMC), anaerobic microbial community (AnMC), and sulphate-reducing microbial community (SO_4_-RMC). The AMC was a group of microorganisms capable of growing in a typical microbiological medium (MH) in the presence of oxygen, while the AnMC and SO_4_-RMC were groups of microorganisms capable of growing in the absence of oxygen. The difference between the AnMC and SO_4_-RMC is the medium used: while the AnMC was grown in a typical microbiological medium (MH), SO_4_-RMC were grown in a selective medium for SRB (*Postgate* C). Mercury resistance levels found for these communities were higher for SO_4_-RMC (Table 1).

### 3.2. Mercury Content after Incubation

**Figure 2:**
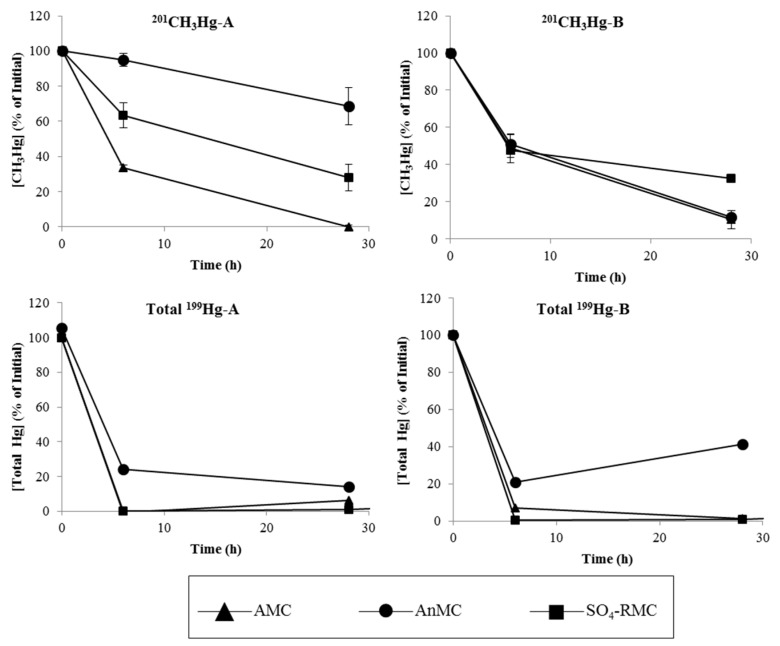
CH_3_^201^Hg and total (all forms) ^199^Hg content in supernatant samples after 6 and 28 h of incubation of three different microbial communities of aerobic microbes (AMC), anaerobic microbes (AnMC), and sulphate-reducing microbes (SO_4_-RMC) after initial addition of 2.05 ng/mL of CH_3_^201^Hg^+^ and 105.6 ng/mL of ^199^Hg^2+^. The three microbial communities were isolated from two areas of the Tagus Estuary in Cala do Norte (**A**) and Barreiro (**B**).

Figure 2 shows the percentage of CH_3_^201^Hg and total ^199^Hg (% of initial) after 6 h and 28 h of incubation in the presence of bacteria. CH_3_^201^Hg decreased over time, with 10–69% remaining after 28 h. Among the three evaluated microbial communities, the highest demethylation rates were registered in AMC samples of isolated communities from Cala do Norte (Figure 2). Total ^199^Hg also decreased over time (Figure 2).

### 3.3. MeHg Formation

**Figure 3:**
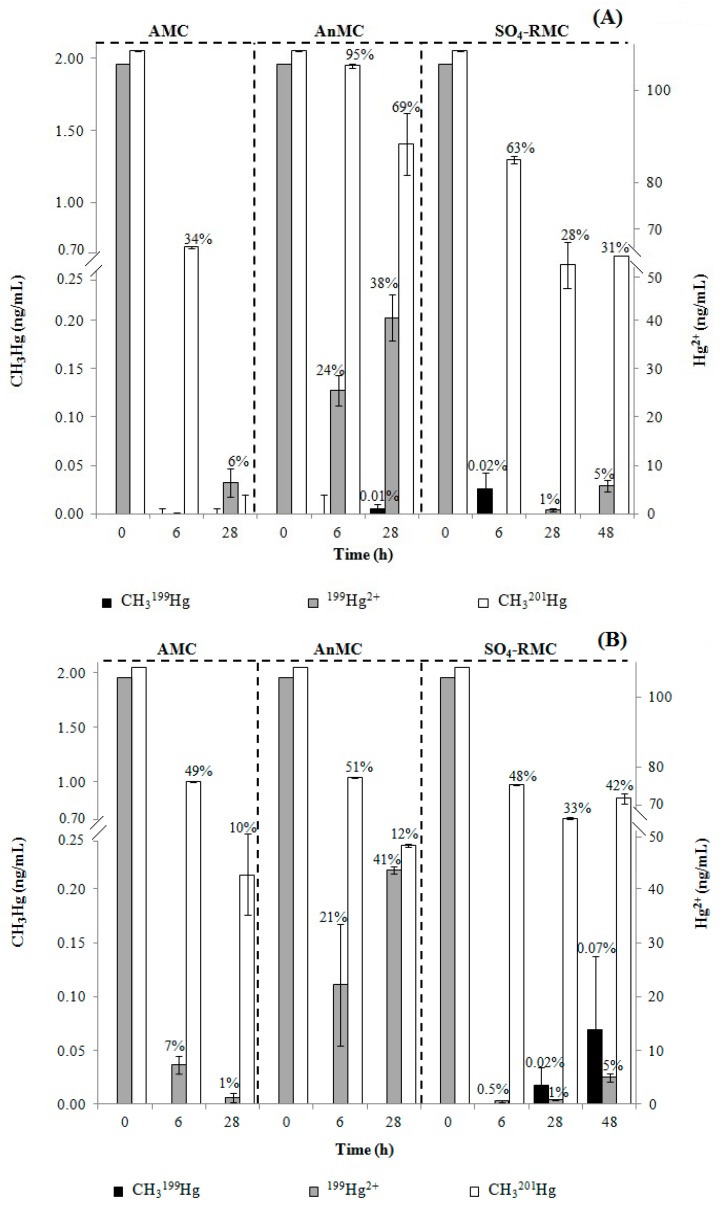
Representation of isotope concentrations in the supernatant of media containing different microbial communities. The results show the formation of CH_3_^199^Hg from the initial 105.6 ng/mL ^199^Hg^2+^ added and the decrease of the initial 2.05 ng/mL of CH_3_^201^Hg with incubation time (6, 28, and 48 h). Three different microbial communities, an aerobic microbial community (AMC), anaerobic microbial community (AnMC), and sulphate-reducing microbial community (SO_4_-RMC), were isolated from two mercury-contaminated areas of the Tagus estuary—Cala do Norte (**A**) and Barreiro (**B**).

Figure 3 shows MeHg concentrations in liquid media samples, discriminating the methylated CH_3_^199^Hg from ^199^HgT. In SO_4_-RMC media, between 0.02% and 0.07% of the initial ^199^Hg (0.02–0.07 ng/mL) was methylated. Methylation was also observed for the AnMC (0.01% of the initial ^199^Hg) (Figure 3).

The highest percentage of methylation was registered in the media containing SO_4_-RMC from Barreiro (Figure 3B).

### 3.4. MeHg Degradation

**Figure 4:**
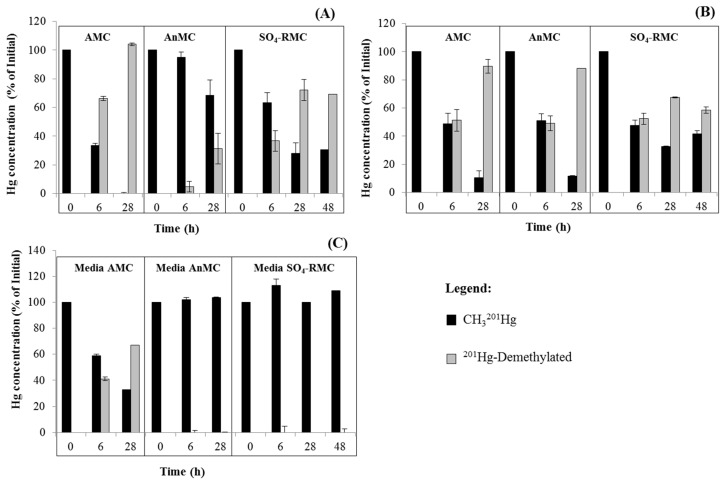
Concentration of CH_3_^201^Hg after 6, 28, and 48 h of incubation with the three microbial communities of aerobic microbes (AMC), anaerobic microbes (AnMC), and sulphate-reducing microbes (SO_4_-RMC) isolated from Cala do Norte (**A**) and Barreiro (**B**). The control media were also evaluated (**C**).

The CH_3_^201^Hg concentration decreased during incubation (Figure 2, Figure 3 and Figure 4). This decrease was more accentuated in media containing the AMC from Cala do Norte, where only 0% to 10% of the initial CH_3_^201^Hg added to media remained after 28 h.

Figure 4 shows the percentage of ^201^Hg demethylated over time. In the noninoculated control, a decrease in CH_3_^201^Hg concentration was also observed for the aerobic control (AMC) as a consequence of abiotic demethylation, albeit to a lesser extent compared to the inoculated homologous sample (Figure 4C). No decrease in CH_3_^201^Hg in the control media of AnMC or SO_4_-RMC was observed (Figure 4C).

### 3.5. Hg^2+^ Reduction and Hg^0^ Volatilization

**Figure 5:**
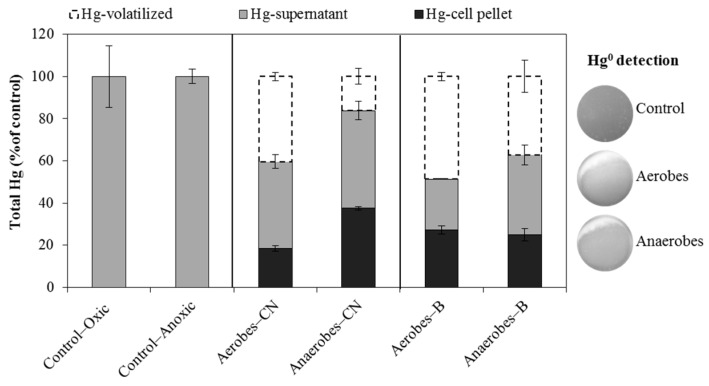
Final balance of total mercury after incubation of aerobic and anaerobic microbial communities isolated from two areas of the Tagus estuary (CN—Cala do Norte and B—Barreiro) with HgCl_2_ for 48 h. The graphic representation shows the percentage of Hg^0^ volatilized and total mercury remaining in the cell pellet and supernatant. Hg^0^ volatilization was detected by the foggy area resulting from the reaction between Hg^0^ and Ag using an X-ray film.

Figure 5 shows that both aerobic and anaerobic communities were able to remove mercury from liquid media containing HgCl_2_ by cell uptake and also by the reduction of Hg^2+^ with subsequent volatilization of Hg^0^. The percentage of reduced Hg^2+^ was 40 and 49% for aerobes and 16 and 37% for anaerobes from Cala do Norte and Barreiro, respectively (Figure 5).

## 4. Discussion

This study shows the role of different communities of bacteria on the processes of mercury methylation and demethylation. The incubation of these communities with isotope-enriched Hg species (^199^HgCl and CH_3_^201^HgCl) highlights their ability to remove both inorganic as well as organomercurial species from the medium (Figure 2). Besides the removal, transformation of inorganic species was also observed, namely their methylation.

Methylation of mercuric mercury (i.e., CH_3_^199^Hg production) was observed in media containing anaerobes, especially SRB (Figure 3). Although methylation was observed previously for a few aerobic bacteria isolated from the Tagus estuary [25], the study of the aerobic community did not detect Hg methylation, suggesting that this type of bacteria does not significantly contribute to MeHg formation.

The highest percentage of observed MeHg formation ranged from 0.02% to 0.07% of ^199^Hg^2+^ initially added (105.6 ng/mL) to the media containing SRB from Cala do Norte and Barreiro, respectively (Figure 3). Likewise, other groups [8,30] also reported that methylation is a process promoted by anaerobes and that over 95% of the mercury methylation occurs in anoxic sediments [31], pointing out sulphate reducers as the main methylators [24,32]. Supplemental Table S1 describes all the isolates for the two studied areas identified during our investigations. These results indicate that the observed methylation by the SO_4_-MRC from Barreiro and Cala do Norte can be related to the presence of *Desulfovibrio desulfuricans*, and for the AnMC from Cala do Norte, can be related to *Clostridium* spp., namely *Clostridium difficile* (Supplemental Table S1). These microorganisms are well known for their methylation potential [31,32,33,34,35,36]; in particular, *Desulfovibrio desulfuricans* ND132 is well known as a model organism for Hg methylation [37]. 

Comparing the two sampled areas, data show that the percentage of methylation was higher in Barreiro (0.07%) than in Cala do Norte (0.02%). Possible explanations for this observation may be differences in bacterial species composition related to the higher and longer-term mercury contamination in Barreiro causing a selective pressure for methylators [7,25]. On average, sediments of Cala do Norte have 11.7 µg/g total Hg, including 28.4 ng/g of MeHg, and sediments of Barreiro have 33.2 µg/g total Hg, including 47.2 ng/g of MeHg [7]. Applying the rate of methylation observed in this study (mentioned above) to ambient field concentrations of total Hg, up to 2.34 ng/g day^−1^ and 11.62 ng/g day^−1^ of MeHg could originate from microbial methylation in Cala do Norte and Barreiro, respectively. It is important to note, however, that physicochemical conditions in the estuary may differ from laboratory conditions. Therefore, these estimates are potential formation rates and still need confirmation in the field.

Demethylation was observed in all inoculated media (Figure 4), indicating that demethylation is common between aerobes and anaerobes [17,34,38] and that both biotic and abiotic mechanisms may be involved [23]. Although *mer B* (organomercurial lyase) was not found among our isolates, reduction of Hg^2+^ into Hg^0^ was detected (Figure 5), which indicates a presence of the *mer* operon amid the isolates; in fact, *mer* A was encompassed by isolates from these areas in our previous study [25]. Thus, we propose that the mechanism for demethylation by microbial communities may also be related to the presence of the *mer* operon. Besides, Figure 3A shows a methylation rate of 0.02% in SO_4_-RMC media after the first 6 h, while no CH_3_^199^Hg was detected after 28 h and 48 h, which could be explained by the simultaneous occurrence of microbial methylation and demethylation processes [33]. Obviously, there is a lot of complexity in this media and certain species may be present, but not sufficiently active, or might be conditioned by the sediment’s chemistry and availability of nutrients [39]. Comparing the proportion of mercury methylation and demethylation in the present study, it appears that in estuarine sediments containing CH_3_Hg^+^ and Hg^2+^, microorganisms are responsible for CH_3_Hg^+^ demethylation in both oxic and anoxic sediments and for methylation in anoxic sediments.

The analyses of ^199^HgT (Figure 2) revealed that Hg^2+^ is removed from liquid media during the incubation with microbes. Between 59–99% of ^199^HgT disappeared after 28 h, which may be explained as a result of cell uptake and reduction of Hg^2+^ to Hg^0^ and its subsequent volatilization from the medium. Both phenomena, i.e., cell uptake and reduction followed by volatilization, were previously observed among individual microorganisms, such as *Bacillus*, *Vibrio*, *Aeromonas*, *Geobacter*, and *Enterobacteriaceae* [7,25], and here also for aerobic and anaerobic communities (Figure 5) isolated from Tagus estuary sediments.

**Figure 6:**
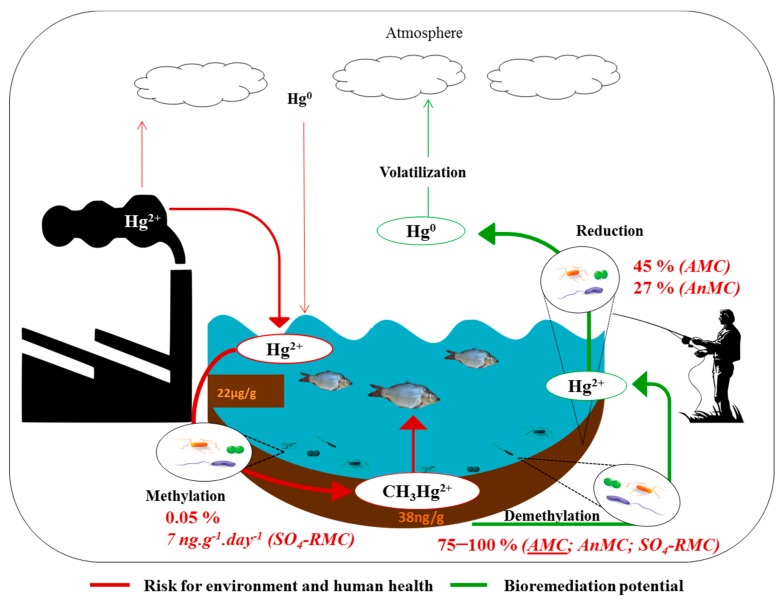
Summary of the results showing microbial-mediated transformations of mercury in the Tagus estuary and their context in human and environmental health. Rate conversions are the averaged values of results reported in this paper for Barreiro and Cala do Norte, and mercury concentrations in sediments, previously reported by Figueiredo et al. [7] were also averaged to indicate the sediments’ levels.

Figure 6 summarizes all the transformations observed by the three evaluated microbial communities of the Tagus estuary, including the simultaneous occurrence of methylation of mercuric mercury and demethylation of MeHg. MeHg demethylation and reduction of mercuric mercury to elemental mercury constitutes a natural detoxification pathway that mitigates the formation and accumulation of the neurotoxic MeHg and promotes the overall removal of mercury from this aquatic system. On the other hand, newly formed MeHg can bioaccumulate, and subsequent biomagnification in food webs represents a severe risk for human and environmental health.

## 5. Conclusions

The Tagus Estuary, one of the largest estuaries in Europe, has a history of high mercury contamination due to past industrial activities. Mercury-resistant bacteria are the main drivers for critical mercury methylation and demethylation processes. Here, we used for the first time isotope-enriched Hg species (^199^HgCl and CH_3_^201^HgCl) for incubations with a diversity of estuarine microbial communities. A significant finding was the existence of concomitant methylation and demethylation processes performed by microbes and the definition of their contribution for the biogeochemical cycle of mercury. Mercury methylation, which represents a risk for environmental and human health, is mainly carried out by SRB in anoxic environments, while significant demethylation was shown for both aerobic and anaerobic microbial communities in anoxic and oxic environments. The latter process may also constitute a potentially important bioremediation pathway. Overall, this study provides important direction for continued investigation of the estuary regarding (i) mercury biogeochemical speciation, (ii) environmental risks, and (iii) wastewater treatment requirements.

## Figures and Tables

**Table 1:** Mercury (HgCl_2_ and CH_3_HgCl) resistance levels of the microbial communities isolated from two mercury-contaminated areas of Tagus estuary—at Cala do Norte and Barreiro.

	HgCl_2_ (µg/mL)			CH_3_HgCl (µg/mL)		
	AMC	AnMC	SO_4_-RMC	AMC	AnMC	SO_4_-RMC
**Cala do Norte**	10	8	50–100	2.5	0.5	2.5
**Barreiro**	13	50	50–100	2.5	10	2.5

**Notes:** AMC: aerobic microbial community, AnMC: anaerobic microbial community, and SO_4_-RMC: sulfate-reducing microbial community.

## Supplementary Materials

The following are available online at http://www.mdpi.com/1660-4601/15/10/2141/s1, Table S1: Isolates identified among the three studied communities. The identifications were based on 16S rRNA amplification and sequencing. Parts of the data were previously published [25].
