# Supplementary material for: Evidence of Mercury Methylation and Demethylation by the Estuarine Microbial Communities Obtained in Stable Hg Isotope Studies
Source: Int J Environ Res Public Health. 2018 Sep 29;15(10):2141. doi: 10.3390/ijerph15102141 (PMC6210349; doi:10.3390/ijerph15102141)
Supplement: Supplementary file 1 [file ijerph-15-02141-s001.pdf]

International Journal of
Environmental Research and Public Health

MDPI

Table S1. Isolates identified among the three studied communities. The identifications were based on 16S rRNA amplification and sequencing.

Microbial communities	Sampled areas	
	**Cala do Norte**	**Barreiro**
**AMC**[a1]	*Bacillus (B. cereus and B. subtilis)*<br>*Serratia marcescens*<br>*Citrobacter freundii*<br>*Pseudomonas putida*<br>*Vibrio metschnikovii*	*Bacillus (B. megaterium, B. cereus, B. subtilis, and B. soli)*<br>*Vibrio (V. metschnikovii and V. fluvialis)*<br>*Aeromonas media)*
**AnMC**[a]	*Clostridium spp.*<br>*Clostridium difficile*<br>*Enterobacteriaceae bacterium*	*Enterobacteriaceae bacterium*
**SO_4-RMC**[a]	*Desulfovibrio desulfuricans*	*Desulfovibrio desulfuricans*

[(a)] AMC: aerobic microbial community, AnMC: anaerobic microbial community, and SO_4-RMC: sulfate-reducing microbial community.

[1] Part of these data were published in Figueiredo et al. [25].
